# Leading reasons for antibiotic prescriptions in pediatric respiratory infections: influence of fever in a primary care setting

**DOI:** 10.1186/s13052-023-01533-5

**Published:** 2023-09-29

**Authors:** Marina Picca, Romeo Carrozzo, Gregorio Paolo Milani, Antonio Corsello, Marina Macchi, Roberto Buzzetti, Paola Marchisio, Chiara Mameli

**Affiliations:** 1 Italian Primary Care Paediatrics Society (SICuPP), Lombardy, Italy; 2 Department of Health Science and Community Health, University of Milan, Milan, Italy; 3 Fondazione IRCCS Ca’ Granda Ospedale Maggiore Policlinico, Milan, Italy; 4 Clinical epidemiologist, Bergamo, Italy; 5 University of Milan, Via della Commenda 9, Milan, 20122 Italy; 6 Department of Pediatrics, Vittore Buzzi Children’s Hospital, Milan, Italy; 7 Department of Biomedical and Clinical Science “L. Sacco”, University of Milan, Milan, Italy

**Keywords:** Respiratory infections, Antibiotic stewardship, Primary pediatric care, Covid-19 pandemic, Drugs over-prescription, Amoxicillin, Children fever phobia

## Abstract

**Background:**

Antibiotic overuse in children is a significant public health concern, as it can lead to the emergence and spread of antibiotic-resistant bacteria. Although respiratory infections account for most antibiotic prescriptions in children, many of these infections are viral and do not require antibiotics. In this study, we aimed to investigate the use of antibiotics in children with respiratory infections in a primary care setting and to explore the possible role of fever on antibiotic prescription.

**Methods:**

We conducted a prospective observational study that evaluated preschool children aged 0–5 years who were assessed by their primary care pediatricians for respiratory infectious diseases between October 2019 and March 2021. The study involved 69 public primary care pediatricians and a total of 678 pediatric episodes for respiratory infections.

**Results:**

Amoxicillin/clavulanate was the most frequently prescribed drug. Bronchitis accounted for most of inappropriate antibiotic prescriptions (73%). Furthermore, the presence of fever was associated with a ~ 300% increase in the likelihood of prescribing antibiotics for respiratory infections that do not typically require antibiotics.

**Conclusion:**

Our findings emphasize the need for adherence to international guidelines and recommendations in the primary care of children to reduce unnecessary antibiotic use and prevent the development of antibiotic resistance. This study also underscores the potential relevance of new studies to evaluate antibiotic prescription attitudes in other clinical settings and geographical areas.

## Background

Respiratory infections are one of the most common diseases affecting children, and primary-care pediatricians typically manage the majority of cases in outpatient settings [1]. The most common reasons for antibiotic prescription in children are Streptococcal pharyngitis, community-acquired pneumonia, sinusitis, and acute otitis media [2]. On the other hand, more widespread infections among children, such as rhinitis, non-streptococcal pharyngitis, bronchitis, and bronchiolitis, typically require only symptomatic management [3–11]. However, inappropriate antibiotic prescriptions frequently occur and are potentially associated with an increase in antibiotic resistance [12].

Fever is a well-established sign of illness, yet it can trigger fear and anxiety in both parents and healthcare providers [13, 14]. Such phenomenon, also known as “fever phobia”, can influence clinical decisions leading to inappropriate care of children with fever [15, 16]. However, the association between fever and antibiotic prescribing in primary care settings has not been extensively investigated. Our study aims to describe the leading reasons for antibiotic prescription in a pediatric population in Italy, before and during the COVID-19 pandemic, in the primary care setting. Furthermore, our study aims to examine the potential correlation between the presence of fever and the inappropriate use of antibiotics in this setting.

## Methods

This prospective observational study was performed between October 2019 and March 2021. A total of 69 primary care pediatricians, members of Italian Primary Care Paediatrics Society (SICuPP) in Lombardy Region (Italy), were involved. The description of their involvement has been recently reported in detail elsewhere [17]. Eligible for the study were all consecutive children aged 0–5 years with a clinically diagnosed acute respiratory infection. Only for Group A streptococcal pharyngitis a rapid antigen test or a cultural test was required for diagnosis. The study excluded episodes of infectious diseases different than respiratory tract infections. Patients with primary immune deficiencies and neurological diseases were not considered eligible for the study, and therefore were excluded. For each patient suspected of a possible respiratory infection pediatricians completed a case report form (CRF) that included the child’s socio-demographic information. The parents were instructed to contact the pediatrician if their child had developed fever (underarm temperature > 38 °C) or became ill. The pediatrician performed a full clinical examination, and if a respiratory infection was suspected, the specific diagnosis was recorded in the CRF, based on the international and statistical classification of diseases and related health problems. The information collected included the patient’s age, sex, diagnosis, antibiotic prescription (if any) and the occurrence of fever at the time of such prescription.

For the descriptive statistics, data are presented as frequency and percentages or median and interquartile range. Missing data were managed with pairwise deletion. Prescriptions of antibiotics for rhinitis, laryngitis, non-streptococcal pharyngitis, bronchitis and bronchiolitis were classified as inappropriate. Chi-Square and Fisher exact tests were used to compare categorical variables. To investigate the association between inappropriate antibiotic prescription and the occurrence of fever, a multiple logistic model was employed. Age and sex were considered for adjustment. A p-value < 0.05 was considered as significant. All analyses were conducted using R-program (2019).

The study was conducted in accordance with the Declaration of Helsinki and was approved in September 2019 by the Ethics Committee of the IRCCS Ca’ Granda Ospedale Maggiore Policlinico Foundation, Milan, Italy.

## Results

Data from 678 pediatric clinical examination conducted for respiratory infections were collected, and 246 antibiotics were prescribed overall (in the 36% of cases). Mean age of the patients was 2.0 ± 1.6 years, 369 of them were male (54% of the total).

The primary reason for antibiotic prescriptions was bronchitis, accounting for 38% of cases, followed by otitis media at 30%. Among these diseases, 56% of bronchitis and 73% of otitis were treated with antibiotics (Table 1). Other infectious disorders, such as rhinitis and sinusitis, which represented 35% of cases in this sample, had a lower rate of antibiotic prescription (< 5%). A summary of the diseases and their corresponding antibiotic prescriptions can be found in Table 1.

**Table 1:** Number of infectious diseases diagnosed and relative antibiotic prescriptions

Disease	Cases (n)	Antibiotic prescriptions (n)	Prescriptions (%)	Fever (%)
Rhinitis	219	7	3.2	27.9
Sinusitis	21	5	23.8	23.8
Non-group A streptococcal pharyngitis	78	20	25.6	70.5
Group A streptococcal pharyngitis	32	32	100	65.6
Otitis media	102	74	72.5	46.1
Laryngitis	41	6	14.6	39
Bronchitis	167	93	55.7	67.3
Bronchiolitis	10	1	10	40
Pneumonia	8	8	100	37.5
**Total**	**678**	**246**	**36.3**	**41.3**

The population was then further analyzed separately considering patients aged 0–2 years (63.9%, n = 433) and patients aged 3–5 years (n = 245). No significant difference emerged in the presence of fever or antibiotic prescription between the two populations (45% vs. 39.2% for fever, 36.3% vs. 38.8% for antibiotic prescription). As expected, no case of bronchiolitis was diagnosed in the 3–5 years age group. Furthermore, among children with pharyngitis, the percentage of Group A streptococcal pharyngitis was higher in 3–5 years-old children compared to the 0–2 years group (9.4% vs. 2.1%, respectively, p < 0.01). Fever (p = 0.035) and antibiotic prescription (p < 0.01) were significantly more frequent in the 0–2 as compared to 3–5 years age group. Fever occurred in 45% in 0–2 and in 39.2% in 3–5 years age group, whereas antibiotics were prescribed in 36.3% in 0–2 and in 38.8% in 3–5 years age group. No further difference was observed between the two age group patients. Out of 246 antibiotic prescriptions, 185 (75%) were available. Main reason for antibiotic prescription was represented by bronchitis (n = 70, 37.8% of total prescriptions retrieved), followed by otitis media (n = 54, 29.2%). These prescriptions, along with their corresponding percentages of the total, are presented in Table 2.

**Table 2:** Antibiotic prescriptions retrieved for single-site infections

Antibiotic	N° of prescriptions	Percentage (%)	Main reason for prescription (%)
Amoxicillin/Clavulanic acid	77	41.6	Bronchitis (37.7)
Amoxicillin	71	38.4	Bronchitis (33.8)
Azithromycin	11	5.9	Bronchitis (72.7)
Cefpodoxime proxetil	8	4.3	Otitis media (75)
Cefixime	5	2.7	Bronchitis (60)
Clarithromycin	5	2.7	Bronchitis (80)
Cefaclor	3	1.6	Bronchitis (66.7)
Cefuroxime	3	1.6	Otitis media (66.7)
Ceftibuten	2	1.1	Group A streptococcal pharyngitis (100)
**Total**	**185**	**100%**	**Bronchitis (37.8)**

Regarding the total number of antibiotic prescriptions, the majority (n = 148, 78%) were for oral administration of amoxicillin: in 48% of cases it was prescribed alone and in 52% in combination with clavulanic acid. Cephalosporins accounted for 11.4% of all antibiotic prescriptions, while macrolides represented 9% of prescriptions.

Among the 678 diagnoses reported, 41.3% of patients presented with fever (n = 280). Out of these patients, up to the 51% of them were prescribed antibiotics, whereas only 26% of non-febrile patients received an antibiotic prescription. In the logistic regression model, the presence of fever was associated with an almost 300% increase in the likelihood of prescribing antibiotics in conditions where it would not generally be recommended (Table 3). Differences in sex and age were not significant.

**Table 3:** Logistic regression of predictive variables associated with the inappropriate prescription of antibiotics for rhinitis, non-group A pharyngitis, laryngitis, bronchitis, and bronchiolitis

	Odds ratio	95% confidence interval	p-value
Age (years)	1.090	0.958–1.240	0.191
Sex (male)	0.880	0.590–1.240	0.529
Fever (presence)	2.920	1.950–4.350	**< 0.0001**

## Discussion

Our study aimed to describe the leading causes of antibiotic prescription in a pediatric outpatient population in Italy before and during the COVID-19 pandemic and to evaluate the possible role of fever in the primary-care pediatrician’s choice. This study may represent an overview of the pediatric primary care clinical setting during the first phase of the COVID-19 pandemic in Italy, during which a sudden reduction in pediatric antibiotic prescription has been reported [18, 19]. Indeed, the pandemic resulted in a change in pediatric healthcare service utilization, with a significant reduction (50%) observed in pediatric clinic visits and hospitalizations, primarily for respiratory diseases [14, 20]. The seasonal pattern of other respiratory viruses, including respiratory syncytial virus and influenza viruses, also markedly varied worldwide [21–23]. Moreover, pediatric antibiotic use declined during the first period of the COVID-19 pandemic compared to the same period in previous years [14, 20]. In particular, a reduction by ~ 80% in the number of prescriptions at primary care pediatric visits for respiratory tract infection was observed in 2020 and 2021 [24–26].

Our results found that bronchitis was the main respiratory reason for antibiotic prescription, followed by otitis media. Indeed, this study points out that healthcare providers should allocate specific attention to the topic of inappropriate prescription of antibiotics for respiratory infections, especially acute bronchitis, which generally only requires supportive treatment [3]. Even if the inclusion of clinical diagnoses of bronchopneumonia in some cases of bronchitis may have been observed, the absence of national guidelines for bronchitis may contribute to an overall increased antibiotic prescribing by pediatricians. Moreover, the persisting misuse of antibiotics in both children and adults poses a significant public health threat, resulting in the proliferation and transmission of antibiotic-resistant bacteria, which represent a critical public health issue in settings such as neonatal intensive care unit, and result in increased morbidity and mortality [27, 28].

In our population fever was a prevalent symptom, and its presence was significantly associated with an almost 300% increase in antibiotic prescription. As fever is a common symptom in childhood and is often linked with viral infections [29], it cannot generally serve as a criterion for prescribing antibiotics [30–32]. We speculate that “fever phobia” could potentially contribute to increasing the prescription of antibiotics within different care settings, from primary care to emergency rooms and intensive cares, along with the spread of antibiotic-resistant bacteria [28, 33, 34]. As an example, persistent fever in case of rhinosinusitis or acute otitis media has often been considered a parameter for defining the severity of the disease in accordance with current guidelines, and has therefore been considered a criterion for antibiotic prescription [35]. Probably, the reduction in the use of Point-Of-Care-Tests (such as pharyngeal T testing for SBEGA, C-reactive protein, complete blood count, and leukocyte formula) due to pandemic-related restrictions may have contributed to increased difficulty in discriminating between bacterial and viral forms of infection, resulting in increased prescription of antibiotics [36, 37]. Among factors affecting the decision of antibiotic prescription, fever plays a decisive role. Chronic disorders may also be among the main further determinant conditions. Some diseases such as immune deficiencies, congenital defects (especially of the airways) or neurological disorders might have played a relevant role in the therapeutical approach, increasing the use of antimicrobial agents.

Amoxicillin/clavulanate has been identified as the most commonly prescribed pediatric antibiotic in primary care settings, consistent with findings from similar studies [25]. Despite being the first-line treatments for many bacterial infections, it is important to always conduct a case-by-case assessment when selecting the association or not of clavulanate with amoxicillin, considering the age of the child, the clinical conditions and the previous antibiotic use [38–40]. Moreover, the use of an effective communication by healthcare providers might reduce caregivers’ anxiety and contribute to a reduction of antibiotic request [41]. Similarly, an effective collaboration among healthcare providers could avoid inconsistent information to caregivers, favorite the adherence to care program and the prescription compliance. These aspects might be more and more relevant in the near future considering the risk of an increase in respiratory viral infections due to the immune debt contracted during the pandemic.

The main strength of the present study lies in its large sample size, evaluated prospectively immediately before and during most of the pandemic period. However, some limitations should be acknowledged: we did not collect information on the length of the fever before antibiotic prescriptions, the investigation was limited to respiratory diseases and data on the type of antibiotic prescribed were not available in all cases. Furthermore, we did not collect data on the occurrence of chronic diseases. Finally, reasons leading primary care pediatricians to prescribe antibiotics were not investigated in detail.

## Conclusions

Our study provides insights into the prescription patterns of antibiotics in a pediatric outpatient population and on the possible role of fever in increasing such attitude. The excessive prescription of antibiotics for some respiratory infections, particularly bronchitis, emphasizes the importance of adhering to international guidelines and promoting antibiotic stewardship in the primary care setting. An inappropriate use of antibiotics may affect future treatment options for patients and pose a threat to public health, due to the increasing issue of antibiotic resistance. The overall reduction in the number of antibiotics prescribed during the pandemic provides an opportunity to reflect on prescribing practices and to promote more appropriate antibiotic use.

## Data Availability

Data and written consents are available from the corresponding author upon reasonable request.

## References

[1] Nash DR, Harman J, Wald ER, Kelleher KJ (2002). Antibiotic prescribing by primary care physicians for children with upper respiratory tract infections. Arch Pediatr Adolesc Med.

[2] Chiappini E, Mazzantini R, Bruzzese E, Capuano A, Colombo M, Cricelli C (2014). Rational use of antibiotics for the management of children’s respiratory tract infections in the ambulatory setting: an evidence-based consensus by the italian society of preventive and social pediatrics. Paediatr Respir Rev.

[3] Hersh AL, Jackson MA, Hicks LA, American Academy of Pediatrics Committee on Infectious Diseases (2013). Principles of judicious antibiotic prescribing for upper respiratory tract infections in pediatrics. Pediatrics.

[4] Wald ER, Applegate KE, Bordley C, Darrow DH, Glode MP, Marcy SM (2013). Clinical practice Guideline for the diagnosis and management of Acute Bacterial Sinusitis in Children aged 1 to 18 years. Pediatrics.

[5] Robinson JL (2021). Paediatrics: how to manage pharyngitis in an era of increasing antimicrobial resistance. Drugs Context.

[6] Fashner J, Ericson K, Werner S (2012). Treatment of the common cold in children and adults. Am Fam Physician.

[7] Manti S, Staiano A, Orfeo L, Midulla F, Marseglia GL, Ghizzi C (2023). UPDATE – 2022 italian guidelines on the management of bronchiolitis in infants. Ital J Pediatr.

[8] Pediatric Outpatient Treatment Recommendations | Antibiotic Use | CDC [Internet]. 2021 [cited 2023 Mar 6]. Available from: https://www.cdc.gov/antibiotic-use/clinicians/pediatric-treatment-rec.html.

[9] Macfarlane J, Holmes W, Gard P, Thornhill D, Macfarlane R, Hubbard R (2002). Reducing antibiotic use for acute bronchitis in primary care: blinded, randomised controlled trial of patient information leaflet. BMJ.

[10] Smith SM, Fahey T, Smucny J, Becker LA (2017). Antibiotics for acute bronchitis. Cochrane Database Syst Rev.

[11] Walsh EE. Acute Bronchitis. Mandell, Douglas, and Bennett’s Principles and Practice of Infectious Diseases. 2015;806–809.e1.

[12] Principi N, Esposito S (2016). Antimicrobial stewardship in paediatrics. BMC Infect Dis.

[13] Barbieri E, di Chiara C, Costenaro P, Cantarutti A, Giaquinto C, Hsia Y (2021). Antibiotic prescribing patterns in Paediatric Primary Care in Italy: findings from 2012–2018. Antibiot (Basel).

[14] Givon-Lavi N, van der Danino D, Sharf A, Greenberg D, Ben-Shimol S (2022). Disproportionate reduction in respiratory vs. non-respiratory outpatient clinic visits and antibiotic use in children during the COVID-19 pandemic. BMC Pediatr.

[15] Merlo F, Falvo I, Caiata-Zufferey M, Schulz PJ, Milani GP, Simonetti GD (2023). New insights into fever phobia: a pilot qualitative study with caregivers and their healthcare providers. Eur J Pediatr.

[16] Clericetti CM, Milani GP, Bianchetti MG, Simonetti GD, Fossali EF, Balestra AM (2019). Systematic review finds that fever phobia is a worldwide issue among caregivers and healthcare providers. Acta Paediatr.

[17] Mameli C, Picca M, Buzzetti R, Pace ME, Badolato R, Cravidi C (2022). Incidence of acute respiratory infections in preschool children in an outpatient setting before and during Covid-19 pandemic in Lombardy Region, Italy. Ital J Pediatr.

[18] Nymand CR, Thomsen JL, Hansen MP. Changes in antibiotic prescribing patterns in danish General Practice during the COVID-19 pandemic: a Register-Based study. Antibiotics. Volume 11. Multidisciplinary Digital Publishing Institute; 2022. p. 1615. 10.3390/antibiotics11111615 PMC9686554 36421259

[19] Kitagawa D, Kitano T, Furumori M, Suzuki S, Shintani Y, Nishikawa H (2023). Impact of the COVID-19 pandemic and multiplex polymerase chain reaction test on outpatient antibiotic prescriptions for pediatric respiratory infection. PLoS ONE.

[20] King LM, Lovegrove MC, Shehab N, Tsay S, Budnitz DS, Geller AI et al. Trends in U.S. outpatient antibiotic prescriptions during the COVID-19 pandemic. Clin Infect Dis. 2020;ciaa1896. 10.1093/cid/ciaa1896 PMC7799289 33373435

[21] Angoulvant F, Ouldali N, Yang DD, Filser M, Gajdos V, Rybak A (2021). Coronavirus disease 2019 pandemic: Impact caused by School Closure and National Lockdown on Pediatric visits and admissions for viral and nonviral Infections-a Time Series Analysis. Clin Infect Dis.

[22] Stera G, Pierantoni L, Masetti R, Leardini D, Biagi C, Buonsenso D (2021). Impact of SARS-CoV-2 pandemic on Bronchiolitis Hospitalizations: the experience of an italian Tertiary Center. Child (Basel).

[23] Tang JW, Bialasiewicz S, Dwyer DE, Dilcher M, Tellier R, Taylor J (2021). Where have all the viruses gone? Disappearance of seasonal respiratory viruses during the COVID-19 pandemic. J Med Virol.

[24] Perrella A, Fortinguerra F, Pierantozzi A, Capoluongo N, Carannante N, Lo Vecchio A (2023). Hospital Antibiotic Use during COVID-19 pandemic in Italy. Antibiot (Basel).

[25] Barbieri E, Liberati C, Cantarutti A, Di Chiara C, Lupattelli A, Sharland M (2022). Antibiotic prescription patterns in the Paediatric Primary Care setting before and after the COVID-19 pandemic in Italy: an analysis using the AWaRe Metrics. Antibiot (Basel).

[26] Dutcher L, Li Y, Lee G, Grundmeier R, Hamilton KW, Gerber JS (2022). COVID-19 and Antibiotic Prescribing in Pediatric Primary Care. Pediatrics.

[27] Serwecińska L, Antimicrobials A-R, Bacteria. A risk to the Environment and to Public Health. Water. Volume 12. Multidisciplinary Digital Publishing Institute; 2020. p. 3313.

[28] Tzialla C, Borghesi A, Serra G, Stronati M, Corsello G (2015). Antimicrobial therapy in neonatal intensive care unit. Ital J Pediatr.

[29] Sullivan JE, Farrar HC, Section on Clinical Pharmacology and Therapeutics, Committee on Drugs (2011). Fever and antipyretic use in children. Pediatrics.

[30] El-Radhi AS. Fever in Common Infectious Diseases. Clin Man Fever Child. 2019;85–140.

[31] Rose E (2021). Pediatric Fever. Emerg Med Clin North Am.

[32] Leekha S, Terrell CL, Edson RS (2011). General Principles of Antimicrobial Therapy. Mayo Clin Proc.

[33] Milani GP, Corsello A, Fadda M, Falvo I, Giannì ML, Marseglia GL (2023). Perception, knowledge and attitude towards childhood fever: a survey among final-year medical students. Br J Clin Pharmacol.

[34] Tersigni C, Montagnani C, D’Argenio P, Duse M, Esposito S, Hsia Y (2019). Antibiotic prescriptions in italian hospitalised children after serial point prevalence surveys (or pointless prevalence surveys): has anything actually changed over the years?. Ital J Pediatr.

[35] Marchisio P, Bortone B, Ciarcià M, Motisi MA, Torretta S, Castelli Gattinara G (2019). Updated Guidelines for the management of Acute Otitis Media in Children by the italian society of pediatrics: Prevention. Pediatr Infect Dis J.

[36] Antoñanzas F, Juárez-Castelló CA, Rodríguez-Ibeas R (2021). Using point-of-care diagnostic testing for improved antibiotic prescription: an economic model. Health Econ Rev.

[37] Lucien MAB, Canarie MF, Kilgore PE, Jean-Denis G, Fénélon N, Pierre M (2021). Antibiotics and antimicrobial resistance in the COVID-19 era: perspective from resource-limited settings. Int J Infect Dis.

[38] Llor C, Bjerrum L (2014). Antimicrobial resistance: risk associated with antibiotic overuse and initiatives to reduce the problem. Ther Adv Drug Saf.

[39] Frieri M, Kumar K, Boutin A (2017). Antibiotic resistance. J Infect Public Health.

[40] Ventola CL (2015). The Antibiotic Resistance Crisis. P T.

[41] Serra G, Giuffrè M, Piro E, Corsello G (2021). The social role of pediatrics in the past and present times. Ital J Pediatr.

